# Electronic Cigarettes on Hospital Campuses

**DOI:** 10.3390/ijerph13010087

**Published:** 2015-12-29

**Authors:** Clare Meernik, Hannah M. Baker, Karina Paci, Isaiah Fischer-Brown, Daniel Dunlap, Adam O. Goldstein

**Affiliations:** 1 Department of Family Medicine, University of North Carolina School of Medicine, Chapel Hill, NC 27599, USA; hmbaker2@unc.edu (H.M.B.); karina_paci@med.unc.edu (K.P.); fiscbro@email.unc.edu (I.F.-B.); adam_goldstein@med.unc.edu (A.O.G.); 2 University of North Carolina School of Medicine, Chapel Hill, NC 27599, USA; daniel_dunlap@med.unc.edu

**Keywords:** electronic cigarettes, smoke-free policy, tobacco products

## Abstract

Smoke and tobacco-free policies on hospital campuses have become more prevalent across the U.S. and Europe, de-normalizing smoking and reducing secondhand smoke exposure on hospital grounds. Concerns about the increasing use of electronic cigarettes (e-cigarettes) and the impact of such use on smoke and tobacco-free policies have arisen, but to date, no systematic data describes e-cigarette policies on hospital campuses. The study surveyed all hospitals in North Carolina (*n* = 121) to assess what proportion of hospitals have developed e-cigarette policies, how policies have been implemented and communicated, and what motivators and barriers have influenced the development of e-cigarette regulations. Seventy-five hospitals (62%) completed the survey. Over 80% of hospitals reported the existence of a policy regulating the use of e-cigarettes on campus and roughly half of the hospitals without a current e-cigarette policy are likely to develop one within the next year. Most e-cigarette policies have been incorporated into existing tobacco-free policies with few reported barriers, though effective communication of e-cigarette policies is lacking. The majority of hospitals strongly agree that e-cigarette use on campus should be prohibited for staff, patients, and visitors. Widespread incorporation of e-cigarette policies into existing hospital smoke and tobacco-free campus policies is feasible but needs communication to staff, patients, and visitors.

## 1. Introduction

Tobacco use remains the leading preventable cause of premature disease and death worldwide, affecting nearly every organ and increasing the risk of multiple forms of cancer, diabetes, tuberculosis, decreased immune function, and resulting in overall diminished health [1,2]. Secondhand smoke produced by tobacco significantly increases the risk of stroke in adults and lung problems in children [3]. Policies to decrease tobacco use and secondhand smoke exposure have been implemented in many diverse settings, including public and private worksites, restaurant and bars, and educational venues [4,5].

Implementing smoke and tobacco-free hospital policies promotes smokers’ attempts to quit and supports availability of local tobacco cessation services [6,7,8]. Bans on hospital smoking and tobacco use also lead to improved rates of smoking cessation among hospital employees, help to de-normalize smoking, and standardize hospital grounds as a non-smoking environment [6,9,10,11]. Initial efforts to incorporate tobacco policies within healthcare environments in the United States focused on voluntary policy changes [10,12,13], followed by a mandate from the Joint Commission on Accreditation of Healthcare Organizations (JCAHO) in 1992 that banned indoor smoking in U.S. hospitals [14]. A sizable minority of hospitals (43%) adopted policies that exceeded the JCAHO requirements by expanding the ban of smoking and/or tobacco use to the entire hospital campus [14]. European countries have also recognized the importance of creating smoke-free health care environments; in 2000, the European Network of Smoke-free Hospitals (ENSH) (now called the Global Network for Tobacco-Free Health Care Services), developed smoke-free policy guidelines for health care facilities [15]. Since then, 1400 hospitals have joined ENSH and implemented tobacco-free campuses, though few have policies extending outdoors [16].

In the U.S., the past decade has indicated a sense of gaining momentum towards establishing 100% smoke and tobacco-free hospital campuses nationwide [17]. In February 2008, JCAHO reported that 45% of hospitals had adopted smoke-free campus policies, and another 15% indicated they would soon implement similar policies [18]. In particular, Arkansas, Wisconsin, North Carolina, and Michigan have emerged as leaders in the development of tobacco-free policies, with 75% or more of their acute care hospitals adopting 100% tobacco-free campus policies [17]. In July of 2015, the American Nonsmokers’ Rights Foundation reported that at least 3844 local and/or state/territory/commonwealth hospitals, health care systems, and clinics had adopted 100% smoke-free campus grounds policies [19]. Despite an exemption of psychiatric and drug-treatment hospitals in the JCAHO smoking ban in hospital buildings [20], the number of psychiatric hospitals prohibiting smoking increased from 48% to 83% between 2008 and 2011 [21], and seven states (Georgia, Michigan, New York, North Carolina, Oklahoma, Vermont, and Virginia) require 100% smoke-free indoor policies at psychiatric facilities [19]. Similarly, the development of tobacco management guidelines specific for mental health services by ENSH has encouraged smoke-free legislation in psychiatric hospitals across Europe [22].

Recently, concerns have arisen about maintaining compliance and effective enforcement of smoke and tobacco-free hospital campus policies, due to the rise in popularity of electronic cigarettes (e-cigarettes) and other electronic nicotine delivery systems [23]. E-cigarettes typically contain nicotine and flavorings, heated to generate a vapor and simulate the act of smoking. Global sales of e-cigarettes reached $6 billion in 2014 [24] with awareness and use of e-cigarettes among adults increasing [25,26,27,28]. Despite the rapid rise in popularity, e-cigarette use remains controversial among the medical and public health community, as long-term data on safety are lacking and efficacy for smoking cessation is limited [25,29]. Specific concerns associated with e-cigarettes include youth access, potential for nicotine addiction, renormalization of a smoking culture, and secondhand exposure of potentially harmful constituents [30,31,32,33]. The diversity of product language (e.g., the distinction between products and what they are called by users) has added to the challenge of accurate surveillance of e-cigarette use and its outcomes [23]; the same is true for effective enforcement of policies labeled as “e-cigarette” policies. 

Policies pertaining to e-cigarette consumption, production, and distribution are in the early stages and vary widely, ranging from no restrictions on producers or consumers to wholesale bans of use and distribution [34]. The European Parliament recently approved a revised Tobacco Products Directive setting regulations for e-cigarettes that ban advertising, require warning labels and childproof packages, and restrict nicotine content [35]. Though e-cigarettes have yet to be regulated in the U.S. [36], the Food and Drug Administration (FDA) has issued warnings concerning potential health risks associated with e-cigarettes [37] and proposed a rule in April 2014 deeming additional tobacco products, including e-cigarettes, to be subject to FDA authority [38]. Confusion exists as to whether e-cigarettes should be treated like other traditional tobacco products or as smoking cessation devices. Research examining how healthcare environments, specifically hospital campuses, have created regulations that address the use of e-cigarettes on the hospital campus is lacking. Additionally, it is unclear what factors motivate the development of such policies or serve as barriers to development, how e-cigarette policies may have been communicated to staff, patients, and visitors, and what level of success hospitals have had in implementing regulations on e-cigarette use.

To explore these issues, this study surveyed hospitals in North Carolina to address the following primary research questions: (1) What proportion of hospitals have created policies explicitly regulating the use of e-cigarettes on campus and where is use regulated (e.g., indoors only or indoors and on grounds); (2) How have e-cigarette policies been implemented, communicated, and enforced; and (3) What factors are motivators for and barriers to the creation of policy regulating use ofe-cigarettes? Results of this study may be useful to hospitals and other institutions across the U.S. and internationally that are currently confronting questions related to implementation of e-cigarette policies.

## 2. Materials and Methods 

### 2.1. Survey Development

Survey items were developed in collaboration with experts in tobacco control policy and tobacco treatment program staff familiar with hospital tobacco-free policies. The survey was finalized after pilot testing among a group of tobacco control researchers to ensure clarity of questions. 

Survey items pertained to: (1) Current policies and use of tobacco products and e-cigarettes at the hospital; (2) Implementation of e-cigarette policies including barriers and motivators to policy development and enforcement and communication of the policy; and (3) Attitudes and perceptions regarding e-cigarette use and policies, including the importance of implementing e-cigarette policies at hospitals, perceptions regarding the extent to which the use of each are a problem on campus among patients, visitors, and staff, and perceptions regarding safety of e-cigarettes for users and bystanders. The web-based survey required approximately ten minutes to complete. All subjects gave their informed consent for inclusion before they participated in the study. The study was conducted in accordance with the Declaration of Helsinki, and the protocol was approved by the University of North Carolina Office of Human Research Ethics (study # 14-1084). 

### 2.2. Participants

All general acute care (*n* = 116) and psychiatric hospitals (*n* = 7) that were listed as members of the North Carolina Hospital Association (https://www.ncha.org/about/member-hospitals) were initially eligible to participate. Each hospital was contacted between April and July 2015. Two acute care hospitals were subsequently determined to be permanently closed at the time of survey, resulting in 121 eligible hospitals. 

The human resource (HR) director or other qualified hospital staff (e.g., wellness coordinator, physician leader) at each hospital was contacted by telephone and/or email and asked to complete a short web-based survey regarding e-cigarette use and policies at the hospital. In each case, the research team attempted to contact the hospital employee who was most qualified to take the survey. No specific questions were used to determine eligibility of respondents. Rather, qualification was determined by the employee having knowledge of an existing tobacco policy, or some type of involvement or interest in implementing a tobacco policy at their hospital, and having a familiarity with enforcement and compliance issues around such policies. Hospital staff were contacted initially by telephone, asking for the person on staff with best knowledge about answering the questions; upon agreement to take the survey, an email with a Qualtrics survey link was sent to that contact. Three reminder emails were sent weekly to participants who had agreed to take the survey but had yet to complete it. 

### 2.3. Demographic Measures

Demographic variables for every hospital were obtained from the American Hospital Association hospital database (updated monthly) in July 2015 [39]. Characteristics included were type of ownership (for-profit, not-for-profit, federal government, or non-federal government), setting (urban or rural), bed size, and American Medical Association (AMA) resident status (teaching or non-teaching).

### 2.4. Data Analysis

SAS version 9.4 (SAS Institute, Cary, NC, USA) was used for data analysis. Differences between survey responders and non-responders in regards to the existence of an e-cigarette policy and demographic variables (*i.e.*, hospital type, ownership, setting, bed size, and AMA resident status) were compared using a χ^2^ or Fisher’s Exact test, with significance at *p* < 0.05. Logistic regression was used to examine the relationship between having an e-cigarette policy and demographic characteristics. 

## 3. Results

A total of 75 North Carolina hospitals completed the survey (response rate of 62%). The non-responding hospitals either declined to take the survey (*n* = 2), agreed to participate but never completed the survey despite reminder e-mails (*n* = 16), completed less than half of thesurvey (*n* = 2), or the research team was not able to get in direct contact with any qualified staff at the hospital (e.g., the research team was referred to a qualified staff member but contact with that person through telephone or email was never able to be made) (*n* = 26). To describe the current prevalence of e-cigarette policies at all North Carolina hospitals, all non-responding hospitals were contacted by phone immediately after the survey was closed and asked whether their hospital had a policy regulating the use of e-cigarettes on campus. Information was successfully collected from 40 of 46 (87%) non-responding hospitals. No statistically significant differences were found between survey responders and non-responders in terms of e-cigarette policy existence, hospital service type, type of hospital ownership, urban or rural setting, bed size, or AMA resident status (Table 1). 

**Table 1:** E-cigarette policies and demographics of NC hospitals.

Hospital Characteristics		Survey Responders (*n* = 75)	Non-Responders (*n* = 46)	*p*-Value *
		% (n)	% (n)	
E-cigarette policy ^**†**^	Yes	81.3% (61)	77.5% (31)	0.62
	No	18.7% (14)	22.5% (9)	
Hospital service type	General acute	94.7% (71)	93.5% (43)	0.79
	Psychiatric	5.3% (4)	6.5% (3)	
Type of ownership	For-profit	10.7% (8)	8.7% (4)	0.72
	Not-for-profit	61.3% (46)	56.5% (26)	
	Federal government	2.7% (2)	4.4% (2)	
	Non-federal government	25.3% (19)	30.4% (14)	
Setting	Urban	52.0% (39)	50.0% (23)	0.83
	Rural	48.0% (36)	50.0% (23)	
Bed size	Small: <100 beds	34.7% (26)	50.0% (23)	0.14
	Medium: 100–299 beds	42.7% (32)	39.1% (18)	
	Large: >299 beds	22.7% (17)	10.9% (5)	
AMA resident status	Teaching	20.0% (15)	8.7% (4)	0.13
	Non-teaching	80.0% (60)	91.3% (42)	

* *p*-value from χ^2^ test or Fisher’s Exact test. ^**†**^ E-cigarette policies from survey non-responders were determined by contacting these hospitals after the survey was closed and asking whether the hospital had a policy regulating the use of e-cigarettes. Policies were obtained from 40 of 46 non-responding hospitals.

### 3.1. Tobacco Use and Policies

**Figure 1:**
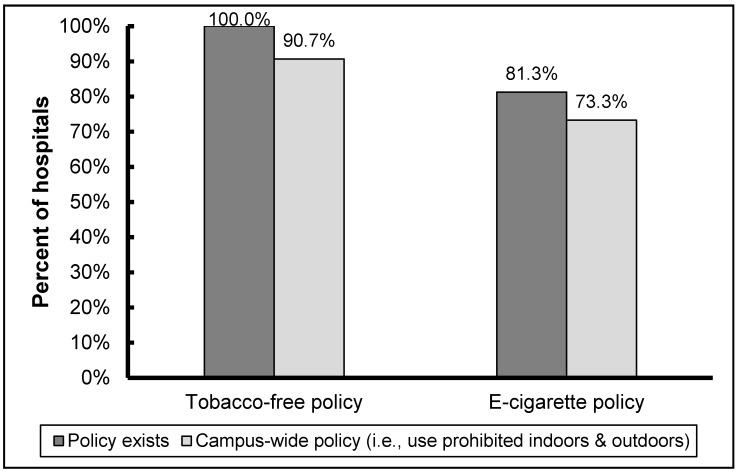
NC hospital campus tobacco policies (*n* = 75).

**Figure 2:**
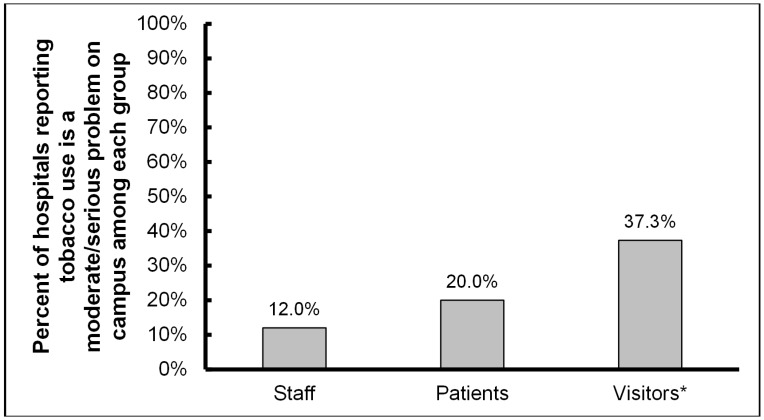
Use of tobacco products currently a problem on the hospital campus (*n* = 75). * Significantly different compared to staff (*p* < 0.01) and patients (*p* = 0.02).

In accordance with the fact that all acute-care hospitals in North Carolina voluntarily became tobacco-free in 2009 [17], all respondents to the current survey reported tobacco-free policies; all psychiatric hospitals in the state also reported being tobacco-free. The majority of survey respondents reported a 100% campus-wide policy (e.g., no designated outdoor smoking shelters) (Figure 1). Though tobacco-free policies have been in place at North Carolina hospitals for multiple years, issues with traditional tobacco use on campus remain. Nearly 40% of hospitals reported a moderate or serious problem with visitors complying with the tobacco-free policy (Figure 2); this problem was significantly greater than compliance issues reported for staff (*p* < 0.01) and patients (*p* = 0.02). 

### 3.2. E-Cigarette Use and Policies

Over four-fifths (81%) of all hospitals reported the existence of a policy regulating the use of e-cigarettes (Figure 1). The overwhelming majority (92%) of the e-cigarette policies reported by survey respondents were campus-wide, with use prohibited indoors and outdoors; use outdoors was limited to certain areas at the remaining hospitals. Of respondents without a policy, nearly half indicated that they were likely to develop a policy within the coming year and most agreed that e-cigarette use among staff, patients, and visitors should be prohibited on campus.

Demographic variables for hospitals reporting an e-cigarette policy were compared to those without an existing policy (Table 2). Hospital setting was the only variable significantly associated with the existence of an e-cigarette policy on campus; the odds of urban hospitals having a policy regulating the use of e-cigarettes were nearly four times greater than rural hospitals (OR = 3.68, CI = 1.33 to 10.19; *p* = 0.01). 

**Table 2:** Predictors of an existing e-cigarette policy (*n* = 115).

Hospital Characteristics		Odds Ratio (95% Confidence Interval)	*p*-Value *
Hospital service type †	Psychiatric	4.13 (0.19–91.14)	0.37
	General acute (ref.)		
Type of ownership	Government	0.52 (0.19–1.45)	0.78
	For-profit	0.36 (0.092–1.41)	0.30
	Not-for-profit (ref.)		
Setting	Urban	3.68 (1.331–0.19)	0.01
	Rural (ref.)		
Bed size	Small: <100 beds	0.21 (0.03–1.78)	0.31
	Medium: 100–299 beds	0.17 (0.02–1.38)	0.11
	Large: >299 beds (ref.)		
AMA resident status	Teaching	2.21 (0.47–10.39)	0.32
	Non-teaching (ref.)		

* *p*-value from logistic regression. † Firth bias-correction used to correct for quasi-separation of data points.

### 3.3. E-Cigarette Policy Implementation 

Nearly all survey respondents with an e-cigarette policy reported that e-cigarettes were added into an existing policy regulating the use of tobacco products, rather than developing a new policy specific to e-cigarettes; policy implementation at two hospitals was unknown. A majority of hospitals (67.2%) with an e-cigarette policy reported that the policy has been somewhat or very effective in reducing the use of e-cigarettes at the hospital. 

Hospitals with and without an existing e-cigarette policy reported the same three primary motivators for the development of a policy: concern that e-cigarettes may be harmful to users; concern that secondhand exposure to e-cigarettes may be harmful or that e-cigarettes may harm indoor air quality; and concern that use of e-cigarettes might renormalize smoking on the hospital campus or lead to increased smoking of traditional cigarettes. No hospitals without an e-cigarette policy reported staff complaints as a motivator for the development of a future policy, whereas 25% of hospitals with existing e-cigarette regulation indicated that complaints from staff motivated them to develop their policy, indicating the importance of staff leadership in promoting policy change.

**Figure 3:**
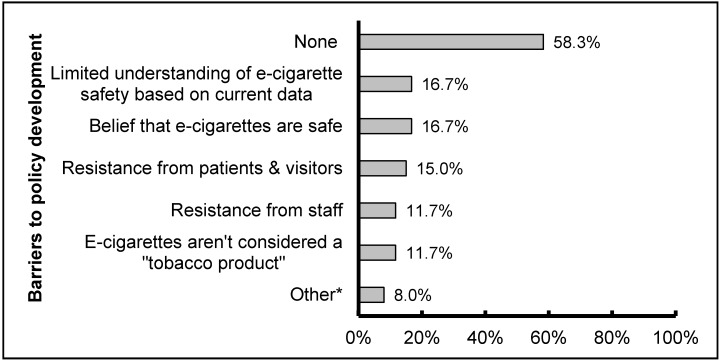
Barriers experienced when developing a policy regulating the use of e-cigarettes (*n* = 60). * Other includes concerns that enforcing e-cigarette policy would be too difficult (3%); legal concerns (3%); and cost of implementation (2%).

A majority (58.3%) of hospitals with an e-cigarette policy reported no barriers in developing that policy. A smaller number reported barriers related to the currently limited state of knowledge about these relatively new products: limited understanding of e-cigarette safety based on current data and belief that e-cigarettes are safe (Figure 3). Similarly, most respondents reported being unsure of the safety of e-cigarettes for users or for those exposed to secondhand vapor or they believed them to be unsafe.

### 3.4. E-Cigarette Policy Communication and Enforcement

**Figure 4:**
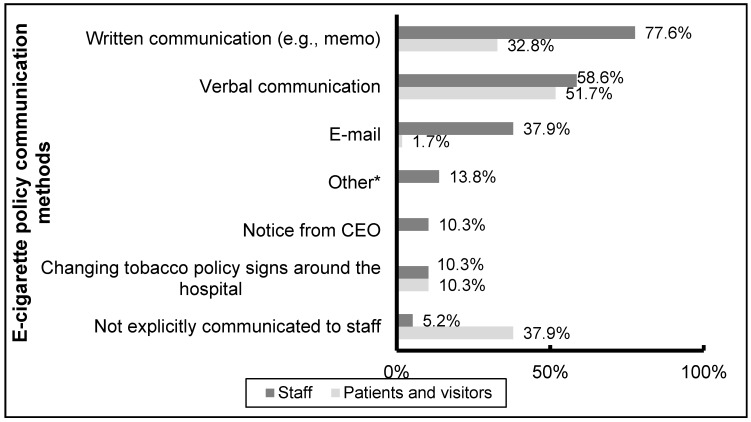
E-cigarette policy communication methods (*n* = 58). * Other includes communication through staff meetings and policy revision updates.

Respondents reported that e-cigarette policies were primarily communicated to staff, patients, and visitors verbally and in written form (e.g., memos) (Figure 4). Only 3 hospital respondents reported that the policy had not been explicitly communicated to staff, though more than one-third reported that the e-cigarette policy had not been explicitly communicated to patients and visitors. A variety of mechanisms were reported as being effective for policy enforcement, primarily encouraging staff to take an active role in policy enforcement (reported by 79% of hospitals) and the use of security (77%) and higher management (49%). 

## 4. Discussion

This study provides the first comprehensive data on the prevalence of e-cigarette regulation on hospital campuses. The overwhelming majority of hospitals in North Carolina have moved towards the development and implementation of e-cigarette policies, with most prohibiting e-cigarette use indoors and outdoors. E-cigarette regulation has primarily been incorporated into existing tobacco-free policies with relatively few reported barriers. Roughly half of the hospitals without a current e-cigarette policy reported that they are likely to develop one within the next year, and most agree that e-cigarette use should be prohibited on campus for staff, patients, and visitors.

Though most hospitals have achieved success in regulating e-cigarettes, the hospital setting was associated with the rate of regulation uptake, with urban hospitals being nearly four times more likely than rural hospitals to have an e-cigarette policy. This differential may involve higher rates of tobacco use in more rural locations [40] or fewer complaints from visitors and staff given the smaller sizes of those hospitals, though accounting for bed size did not affect the association between setting and the existence of an e-cigarette policy. Further examination is needed regarding why e-cigarette regulation at rural hospitals may be lower, including more in-depth exploration of barriers to policy development and implementation and whether this situation generalizes to other rural hospitals throughout the U.S.; still, the majority of rural hospitals in the state (68%) have adopted comprehensive e-cigarette policies. 

While respondents indicated that current hospital policies are effectively limiting major problems with traditional tobacco and e-cigarette products on hospital campuses, moderate problems may still exist, especially concerning compliance among hospital visitors. For e-cigarettes specifically, the lack of policy communication to patients and visitors reported by hospitals likely contributes to greater problems enforcing the expanded policies and confusion about whether e-cigarette use is allowed. Changing existing tobacco policies to include e-cigarettes without communicating these changes (e.g., changing tobacco policy signs around the hospital and notes in patient rooms) offers limited ability to simultaneously regulate the use of tobacco products and e-cigarettes on hospital campuses. Without proper communication and enforcement, e-cigarette use in settings such as hospitals may renormalize smoking behavior and sustain nicotine addiction among tobacco users [30]. 

The strong support for and enactment of policies for tobacco-free hospital campus environments that now includes e-cigarettes will soon involve almost all hospitals in North Carolina, including psychiatric hospitals. This support, in the country’s leading tobacco-producing state, should serve to promote the adoption of comprehensive tobacco and e-cigarette campus policies not only at other hospitals throughout the U.S. and globally, but also in private spaces such as worksites, restaurants, and bars, which currently lack such regulations in many states [31,41,42]. At the same time, more research is needed to guide tobacco control practitioners and policymakers about the effects of e-cigarettes on users and bystanders and the impact on population health in order to inform more effective e-cigarette regulation [43,44]. 

Though the proposed rule that would give the FDA authority to regulate e-cigarettes as a tobacco product has yet to be finalized, action at the state and local level has been taken in accordance with the WHO’s recent report calling for the ban of electronic nicotine delivery systems indoors until it can be proven that exhaled vapor is not harmful to bystanders [45]. The American College of Physicians has issued a similar statement recommending that federal, state, and local policymakers extend indoor and public place clean air laws to these products to prevent secondhand exposure, the renormalization of smoking, and confusion regarding product safety that may result from more widespread use [46]. The ENSH-Global Network of Tobacco-Free Health Care Services and the International Network of Health Promoting Hospitals and Health Services have also recently issued a recommendation to prohibit the use of e-cigarettes indoors and outdoors at all hospitals and health services [47]. As of July 2015, 394 local governments and three states in the U.S. had incorporated e-cigarette restrictions into existing smoke-free laws [48]. These include many large cities, such as Los Angeles, San Francisco, Chicago, and New York City, which have prohibited e-cigarette use any place where smoking is prohibited [48]. Across Europe, smoke-free legislation has begun including e-cigarettes, and countries such as Spain and Belgium have prohibited e-cigarette use in public spaces [49]. Our findings support this growing movement toward regulating e-cigarettes and provide evidence that JCAHO has the opportunity to expand its mandate of smoke-free indoor hospitals in the U.S. to include the use of e-cigarettes. Using the existing momentum from states and national organizations, JCAHO has to opportunity to provide protection from any potential harms of e-cigarette use and secondhand vapor to hospital patients, visitors, and staff. 

### Limitations

Two limitations should be considered when interpreting study findings. First, all survey data were self-reported by respondents at hospitals. Though every effort was made to contact the most qualified staff member to complete the survey, responses are likely influenced by the respondent’s extent of involvement and experience in the development, implementation, communication, and enforcement of hospital policies. Second, survey data may result in non-response bias. However, a sufficiently high response rate was achieved (62%) and no statistically significant differences were found between responders and non-responders in terms of demographics. Hospitals with an existing e-cigarette policy were not more likely to respond than hospitals without a policy, as we obtained policy information from 95% of North Carolina hospitals, helping ensure the validity of survey findings.

## 5. Conclusions

The great majority of North Carolina hospitals have moved forward with regulating e-cigarettes on campus, a finding that may serve as a model for other hospitals and diverse settings across the world confronting questions on how to regulate e-cigarette use in already smoke-free and tobacco-free environments. Incorporating e-cigarette regulation into existing tobacco-free policies occurred with few problems, but issues remain in effectively communicating updated policies to hospital patients and visitors. 

## Abbreviations

The following abbreviations are used in this manuscript:

JCAHO: Joint Commission on Accredidation of Healthcare Organizations

ENSH: European Network of Smoke-free Hospitals

FDA: Food and Drug Administration

AMA: American Medical Association
